# Ocular characteristics and complications in patients with osteogenesis imperfecta: a systematic review

**DOI:** 10.1111/aos.14882

**Published:** 2021-05-19

**Authors:** Sanne Treurniet, Pia Burger, Ebba A.E. Ghyczy, Frank D. Verbraak, Katie R. Curro‐ Tafili, Dimitra Micha, Nathalie Bravenboer, Stuart H. Ralston, Ralph de Vries, Annette C. Moll, Elisabeth Marelise W. Eekhoff

**Affiliations:** ^1^ Department of Internal Medicine, Section Endocrinology Amsterdam Bone Center Amsterdam University Medical Center Amsterdam The Netherlands; ^2^ Department of Ophthalmology Amsterdam University Medical Center Amsterdam The Netherlands; ^3^ Department of Clinical Genetics Amsterdam Movement Sciences Amsterdam University Medical Center Amsterdam The Netherlands; ^4^ Department of Clinical Chemistry, Bone and Calcium Metabolism Lab Amsterdam University Medical Center Amsterdam The Netherlands; ^5^ Centre for Genomic and Experimental Medicine MRC Institute of Genetics and Molecular Medicine University of Edinburgh Edinburgh UK; ^6^ Medical library Vrije Universiteit Amsterdam The Netherlands

**Keywords:** osteogenesis imperfecta, ophthalmology, eye disease, collagen alteration, collagen type I

## Abstract

**Purpose:**

Osteogenesis imperfecta (OI) is a rare inherited heterogeneous connective tissue disorder characterized by bone fragility, low bone mineral density, skeletal deformity and blue sclera. The dominantly inherited forms of OI are predominantly caused by mutations in either the *COL1A1* or *COL1A2* gene. Collagen type I is one of the major structural proteins of the eyes and therefore is the eye theoretically prone to alterations in OI. The aim of this systematic review was to provide an overview of the known ocular problems reported in OI.

**Methods:**

A literature search (in PubMed, Embase and Scopus), which included articles from inception to August 2020, was performed in accordance with the PRISMA guidelines.

**Results:**

The results of this current review show that almost every component of the eye could be affected in OI. Decreased thickness of the cornea and sclera is an important factor causing eye problems in patients with OI such as blue sclera. Findings that stand out are ruptures, lacerations and other eye problems that occur after minor trauma, as well as complications from standard surgical procedures.

**Discussion:**

Alterations in collagen type I affect multiple structural components of the eye. It is recommended that OI patients wear protective glasses against accidental eye trauma. Furthermore, when surgery is required, it should be approached with caution. The prevalence of eye problems in different types of OI is still unknown. Additional research is required to obtain a better understanding of the ocular defects that may occur in OI patients and the underlying pathology.

## Introduction

Osteogenesis imperfecta (OI) is a rare inherited heterogeneous connective tissue disorder characterized by bone fragility, low bone mineral density (BMD) and skeletal deformity. Other clinical findings include blue sclerae, dentinogenesis imperfecta, hyperlaxity of the ligaments, cardiovascular diseases and hearing loss. Although considered a rare disease, OI is one of the most common inherited skeletal dysplasias, with a reported prevalence from 3–10/100 000 (Sillence et al., 1979a, 1979b; Monti et al. 2010; van Dijk et al. 2011; Van Dijk & Sillence 2014; Marini et al. 2017).

**Fig. 1:**
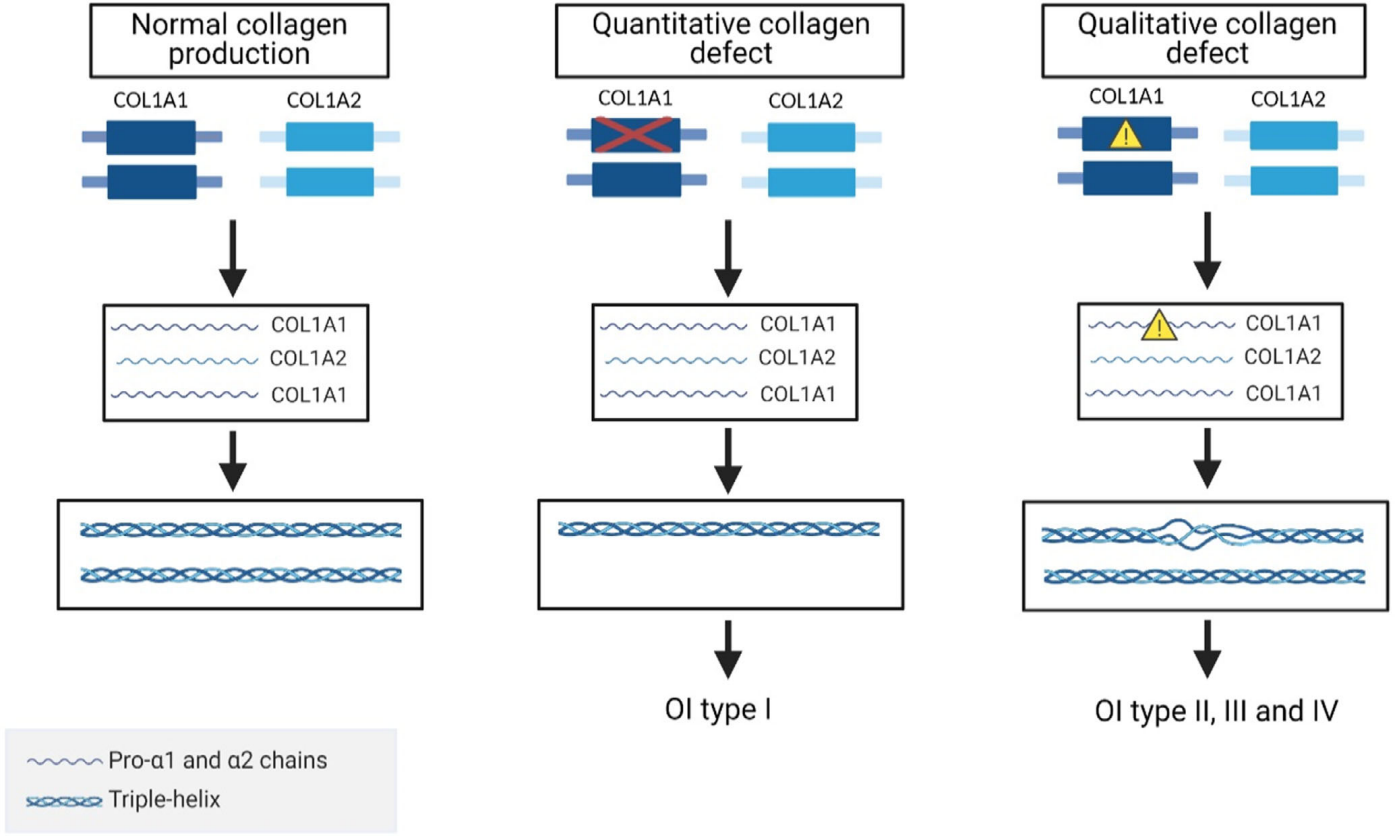
Schematic presentation of *COL1A1* and *COL1A2* genes and the production of collagen type I molecules. *COL1A1* codes for two pro‐α1 chains and *COL1A2* codes for one pro‐α2 chain. Two pro‐α1 and one pro‐α2 chains combine to a triple helix. After extensive post‐translational modifications, the mature collagen type I is formed. A quantitative collagen defect can be produced by gene deletions by which the produced collagen type I is reduced to 50%; this usually leads to mild OI (type I). A quantitative collagen defect can be caused by mutations affecting the collagen type I folding; these usually lead to more severe OI forms (type II, III and IV). Qualitative defects delay the folding of collagen leading to excessive post‐translational modification; this can lead to lower packing density of collagen molecules in the fibril dysregulating mineralization. In the case of quantitative defects, some level of increased overmodification can be also potentially expected due to the increased ratio of collagen‐modifying enzymes to collagen but the consequences of that remain unclear. *Created with BioRender.com*.

In approximately 90% of all cases, OI is inherited as an autosomal dominant trait. The dominantly inherited forms are mainly caused by mutations in either the *COL1A1* or *COL1A2* genes, which encode the α1 or α2 chains of type I collagen respectively (Fig. 1). The collagen type I molecule is a triple helix formed by two α1 and one α2 chains. The synthesis of the collagen type I chains in the ribosomes is accompanied by extensive post‐translational modifications. Once secreted, the N‐ and C‐terminus propeptides are cleaved off which allows the mature collagen molecules to form fibrils. Collagen fibrils are subsequently bundled to form collagen fibres (Forlino & Marini 2015; Nijhuis et al. 2019). 90% of the body’s collagen consists of collagen type I. This is a major structural protein of bone, skin, eyes and other tissues, primarily formed by fibroblasts and osteoblasts. In bone it forms a framework for mineral deposition, which is needed to withstand compression and bending forces (Forlino & Marini 2015; Marini et al. 2017). Individuals with collagen type I mutations can be classified as four defined types of OI. This classification was established by Sillence et al. in 1979 and is based on clinical and radiological findings (Sillence et al. 1979). Osteogenesis imperfecta (OI) type I is generally caused by mutations which lead to failure to synthesize sufficient quantities of type I collagen. This type is the least severe, with a limited number of fractures and deformities. A characteristic finding in these patients is the blue sclera. Mutations causing OI types II‐IV are frequently associated with structural abnormalities of collagen molecules. Osteogenesis imperfecta (OI) type II is lethal and causes death *in utero* or shortly after birth because of severe fractures and pulmonary failure. Patients with OI type III are severely affected and deteriorate from multiple fractures and deformities throughout life. Osteogenesis imperfecta (OI) type IV is relatively mild with a limited number of fractures and blue sclera during childhood. During a consensus meeting in 2009, OI type V was added to the classical Sillence classification (Warman et al. 2011; Van Dijk & Sillence 2014). OI‐V is caused by a mutation in the *IFITM5* gene, affecting bone matrix mineralization. This type is characterized by bone fragility, mineralization of the interosseous membrane and hyperplastic callus formation (Van Dijk et al. 2012).

Skeletal fractures and deformities are the most prominent clinical symptoms of OI. Because of their severity, the attention has been diverted away from other symptoms associated with OI such as dentinogenesis imperfecta, hyperlaxity of the ligaments and hearing loss. The clinical presentation of the other symptoms associated with OI varies significantly among patients; dental abnormalities manifest in only one quarter of patients, and hearing loss is uncommon in children (Lund et al. 1998; Kuurila et al. 2000; Carre et al. 2019). An underinvestigated area in OI is eye‐related problems. Collagen is one of the major structural components of the eye. To date, 29 human collagen types have been identified. The collagen types I‐IX are present in the eye, of which collagen type I is the most abundant one (Sorushanova et al. 2019). Collagens are characterized by the presence of the triple helix which is however found to a variable extent amongst different collagen types. These structural differences can determine their functional properties based on which different types of collagen fibrils are formed. Table 1 shows an overview of collagen types’ distribution in different components in the eye under physiological conditions (Marshall et al. 1993); however, the exact function of the different collagen types and their respective fibrils in the eye remains largely unknown. Considering the variable collagen composition of the different eye structures, a challenging question is also the impact of collagen type I mutations on the quality and functional competence of each structure.

**Table 1:** Overview of distribution of collagen types among the main eye structures

Anatomical structures of the eye		Type of collagen								
		I	II	III	IV	V	VI	VII	VIII	IX
Sclera										
Cornea	Basement membrane									
	Bowman’s membrane									
	Stroma									
	Descemet membrane									
Iris	Stroma									
	Vascular basement membrane									
	Dilator muscle membrane									
Ciliary body	Basement membrane									
	Stroma									
	Muscle									
Lens										
Vitreous body										
Retina	Vascular basement membranes									
	Central artery									
Choroid	Bruch’s membrane									

Collagen type I, II and III and V belong to the fibril‐forming collagens which can combine to form heterotypic fibrils and are important for the structure of the eye. Collagen type V has the ability to anchor basement membranes to the underlying structure. Collagen types IV and VIII are both network‐forming collagens of which the first forms a fibrillar meshwork in the basement membrane of cells. Collagen VII forms anchoring fibrils, important for basement membranes. Collagen type VI and VIII are short‐chain collagen fibrils and support collagen networks. Collagen type VI and VIII are short‐chain collagen fibrils and support collagen networks. Collagen type IX is found to be associated with collagen fibrils, and while it does not form fibrils itself, it is important for the vitreous substance. Black squares indicate the collagen type is present in the indicated component of the eye.

Collagen type I is particularly known for its tensile strength and is primarily seen in the sclera and cornea. The sclera is an opaque tissue, mainly composed of thick and densely packed collagen type I fibrils (90%) to provide a strong outer shell for the eye (Marshall et al. 1993; Coudrillier et al. 2015). The collagen fibres are of various diameter and the spacing between the fibrils is irregular (Coudrillier et al. 2015). Blue discoloration of the sclera is one of the main clinical characteristics of OI. This is caused by thinning of the collagen fibres with increased translucency of the sclera, revealing the underlying choroid. The cornea is also mainly composed of collagen type I (90%) (Bailey 1987). The collagen fibrils of the cornea have a small uniform diameter and are arranged in an orderly fashion to provide transparency (Meek 2009). Furthermore, collagen type I is found in the iris, lens, retina and the Bruch’s membrane. According to current guidelines, examination of the eyes for patients with OI is not a standard procedure. Although the eyes are theoretically prone to alterations as a result of genetic mutations, still little is known about the prevalence and pathophysiology of this problem in OI. Therefore, the aim of this systematic review is to comprehensively describe the type of eye problems reported in patients with OI.

## Methods

### Literature search

A literature search was performed based on the Preferred Reporting Items for Systematic Reviews and Meta‐Analyses (PRISMA)‐statement. To identify all relevant publications, systematic searches were conducted in the bibliographic databases PubMed, Embase.com and Scopus from inception up to 8 August 2020, in collaboration with a medical information specialist.

Search terms included controlled terms from MeSH in PubMed and Emtree in Embase.com in addition to free‐text terms. In Scopus it only included free text. The following terms were used (including synonyms and closely related words): “Osteogenesis Imperfecta”, “Eye Diseases”, “Visually Impaired”, “Visually Disabled”. The search was performed without date or language restrictions. The full search strategies for all databases can be found in the Supplementary Information.

### Study selection

**Fig. 2:**
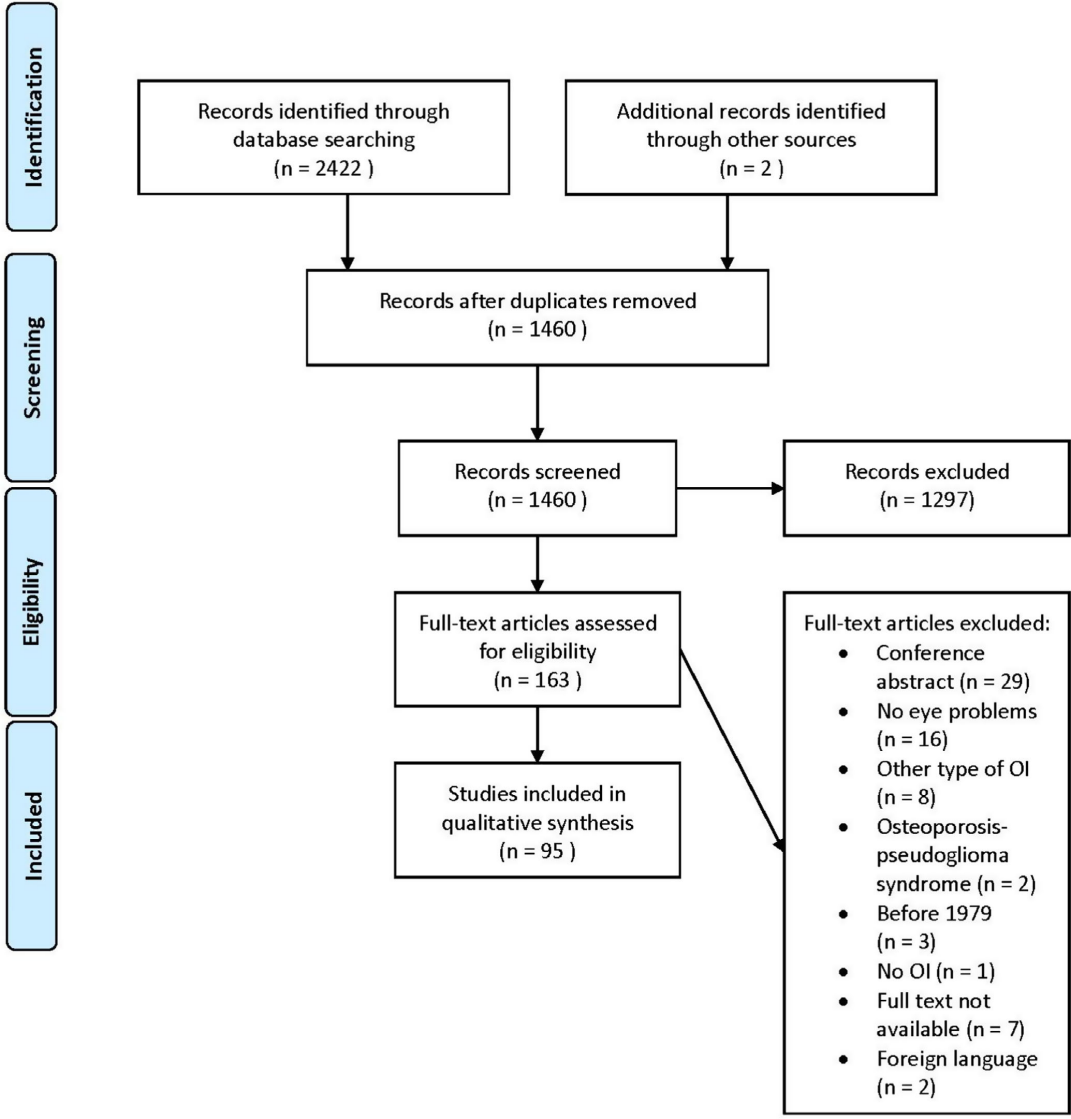
Prisma diagram of study selection process.

After deduplication a total of 1460 papers were identified. First all titles were screened for eligibility by two researchers (ST and PB) after which all remaining abstracts were screened. In case of disagreement consensus was obtained by dialogue. A total of 163 studies were included for full‐text analysis. Sixty‐eight studies were excluded following full‐text analysis (Fig. 2).

### Inclusion criteria

The following inclusion criteria were used: (1) studies were published in either English or Dutch; (2) studies contained patients with osteogenesis imperfecta type I‐V; (3) studies gave an adequate description of the eye problem(s); (4) studies described children and/or adults; (5) studies published as an original article; (6) full text availability and (7) all types of study design.

### Exclusion criteria

The following exclusion criteria were used: (1) studies published before 1979; (2) osteoporosis‐pseudoglioma syndrome (osteogenesis imperfecta ocular form); (3) Cole Carpenter syndrome; and (4) Bruck syndrome.

### Quality assessment

Quality assessment was performed on all articles describing eye problems other than only blue sclera. The Quality assessment was independently conducted by two researchers (ST and PB). The vast majority of the articles consisted of case reports (64%), and the quality of the reports was rated using the method described by Murad et al. (2018). The remaining 36% consisted of case‐control, cohort, cross‐sectional studies and case‐series. These articles were assessed using the Study Quality Assessment Tool created by NHLBI.

## Results

A total of 95 studies were included in this systematic review. Results of the quality assessment identified 62% of the studies as being of good quality, 30% of fair quality and the remaining 8% of poor quality. Due to the low number of articles describing eye problems, no articles were excluded on the basis of the quality assessment. An overview of the included studies and quality rating can be found as a supplement. This section describes all ocular findings in OI categorized in findings possibly associated with OI and incidental findings. Table 2 shows the number of published articles per anatomical location of the eye.

**Table 2:** Overview of number of published articles describing eye defects in OI patients per anatomical location.

	Eye defects							
	Muscles	Cornea	Sclera	Macula	Retina	Choroidea	IOP/Glaucoma	Refraction
Number of articles	1	22	54	2	8	4	6	18

### Findings possibly associated with OI

#### 1. Cornea

##### 1a. Histology

In a post‐mortem case series from Chan et al. (1982) histological findings of the eyes of four patients with OI type II were described. They showed that the thickness of the collagen fibres in the cornea was reduced by 25% compared with a 3‐months old child who died of a non‐connective tissue disorder. Furthermore, the collagen fibres in the sclera were reduced by more than 50%. This series revealed that the collagen fibres did not demonstrate the typical cross striation and had the more translucent appearance of immature collagen. Lastly, the fibres were shown to be more uniform and to have a smaller range of differences in their diameter.

##### 1b. Corneal thickness

Several studies performed central corneal thickness (CCT) measurements (Table 3) (Kaiser‐Kupfer et al., 1981a, 1981b; Pedersen & Bramsen 1984; Kaiser‐Kupfer et al., 1985a, 1985b; Kasner et al. 1992; Evereklioglu et al. 2002; Liu et al. 2007; Dimasi et al. 2010; Kobayashi et al. 2014; Wallace et al. 2014; Bohnsack et al. 2016; Sendul et al. 2016; Kwitko & Pretto 2017; Zeri et al. 2017; Bellanca et al. 2018; Hald et al. 2018; Koyun et al. 2018; Lagrou et al. 2017; Magalhaes et al. 2018; Keleş et al. 2020). Seven studies showed that the mean CCT in patients with OI was significantly lower than in healthy controls (Pedersen & Bramsen 1984; Evereklioglu et al. 2002; Dimasi et al. 2010; Hald et al. 2018; Lagrou et al. 2017; Magalhaes et al. 2018; Keleş et al. 2020). Dimasi et al. (2010) conducted a genetic study where one SNP in *COL1A1* and three SNPs in *COL1A2* were associated with normal CCT variation in the normal population. This implies that collagen type I is a determinant of CCT in both OI patients as well as healthy individuals. Evereklioglu et al. (2002) described the correlation between blue sclera and CCT: eyes of OI patients with blue sclera (OI type I) had significant lower CCT readings when compared with eyes without blue sclera from OI patients. This relation between CCT and blue sclera was also confirmed by Hald et al. (2018). They also showed that patients with OI type I had a mean CCT of 461 ± 32 μm, which was significantly lower than patients with OI type III and OI type IV, with CCT values of 510 ± 29 μm and 500 ± 40 μm respectively. The authors stated that this correlates with the underlying collagen pathology: CCT values are markedly lower in OI type I patients, that have a quantitative collagen defect, compared to patients with a qualitative defect (OI type III and IV). Patients with OI type III and IV have CCT values below individuals without OI, but not as low as the CCT levels in patients with an isolated quantitative defect. However, Koyun et al. (2018) reported no statistically significant difference in CCT values between OI type I and OI type IV. Nonetheless, the authors stated that the small sample size of 15 patients should be taken into account.

**Table 3:** CCT measurements in OI patients.

Article	Participants (*n*)		OI‐type	Age (Y/M)	CCT (µm)	
	OI	Controls			OI	Controls
Everegklioglu et al. 2002	23	15	I = 12 IV = 11	OI = 10.1 ± 2.5Y Controls = 9.8 ± 1.8Y	495.5 ± 24.6	543.6 ± 21.4*
					OI‐I versus OI‐IV = 446.5 ± 16.3 versus 473.6 ± 25.0*	
Dimasi et al. 2010	28	949	I	OI = 34.1Y	450.7 ± 42.8	539.6 ± 32.7*
Hald et al. 2018	64	123	I = 45 III = 7 IV = 13	OI = 44.9 ± 15.9Y	OI‐I 461 ± 32 OI‐III 510 ± 29 OI‐IV 500 ± 40	550 ± 30
Koyun et al. 2018	15	‐	I = 8 IV = 5 UN = 2	15.3 ± 5.4Y	Mean 492 ± 67.49 OI‐I 461 ± 44 OI‐IV 508 ± 79	‐
Magalhaes et al. 2018	42	42	I = 20 III = 6 IV = 16	21.7 ± 2.5Y	OI‐I 443.7	543.9*
					OI‐III 505.1	541.9*
					OI‐IV 496.3	548.6*
Keles et al. 2020, ^†^	17	19	I = 3 III = 11 IV = 3	OI = 14.3 ± 5.4Y Controls = 12.6 ± 4.4Y	482.5 ± 66.9	556.4 ± 37.7*
Lagrou et al. 2017	10	30	I = 7 (4 with blue sclera) UN = 3	OI = 13.7 ± 4.3Y Controls = 12.7 ± 2.5Y	449.8 ± 30.8	568.6 ± 47.6*
					blue versus white sclera = 437.5 ± 23.3 versus 458 ± 30.2	
Pedersen & Bramsen 1984	53	Otosclerosis = 35 Controls = 35	‐	OI = 35Y Otosclerosis = 47Y Controls = 47Y	443 ± 3	Otosclerosis 515 ± 4*
					443 ± 3	552 ± 4*
Kaiser‐Kupfer et al. 1981	16	16	‐	Range: 6–56Y	580 ± 110	580 ± 20
Kaiser‐Kupfer et al. 1985	45	51	‐	OI = 28.7 ± 15.3Y	570 ± 100	570 ± 20
Bellanca et al. 2018	1	‐	I	28Y	460 OD/454 OS	‐
Bohnsack 2016	1	‐	‐	6 M	436 OD/282 OS	‐
Kasner et al. 1992, ^≠^	1	‐	III	18Y	480 OU	‐
Kobayashi et al. 2014	2	‐	I	67Y 26Y	386 OD/384 OS 430 OD/425 OS	‐
Kwitko & Pretto 2017	1	‐	‐	6Y	445 OD/420 OS	‐
Liu et al. 2007	1	‐	I	14Y	434 OD/441 OS	‐
Sendul et al. 2016	1	‐	‐	12Y	500 OD/510 OS	‐
Wallace et al. 2014	3	‐	I	74Y 70Y 49Y	377 OD/374 OS 426 OD/432 OS 452 OU	‐
Zeri et al. 2017	2	‐	I	31Y 4Y	531 OU 505 OD/507 OS	‐

OI = osteogenesis imperfecta, CCT = central corneal thickness (mean ± SD), *n* = number, Y = years, M = months, UN = unknown.

* Statistical significant.

^†^ Only right eye examined.

^≠^ Post‐mortem examination.

Kaiser‐Kupfer et al., (1981a, 1981b) showed a significant lower ocular rigidity in 16 OI patients compared to age, sex and refraction matched controls. They also showed a significantly smaller corneal diameter and axial length of the globe indicating that eyes in OI patients were significantly smaller compared to controls (22.8 mm versus 24.0 mm). Later, Kaiser‐Kupfer et al., (1985a, 1985b) demonstrated in a cohort of 50 OI patients the correlation of decreased ocular rigidity with increasing blueness of the sclera. Surprisingly, a difference in corneal thickness between patients and controls was not confirmed.

##### 1c. Keratoconus

Magalhaes et al. (2018) performed a case–control study in 42 OI patients to evaluate corneal profiles. Corneal pachymetry was significantly thinner in all corneal positions for OI (type I, III and IV) compared to matched controls. No statistical significant differences in anterior and posterior radii of curvatures were found and the authors concluded that keratoconus does not occur frequently in OI.

Keleş et al. (2020) reported on anterior segment findings in 17 eyes of patients with OI compared to age‐matched healthy control subjects. The main findings with statistical significance were thinner central corneal thickness, significantly higher front corneal astigmatism, higher index of vertical asymmetry (IVA), index of height asymmetry (IHA), higher posterior elevation (PE) and higher mean D value in BAD‐III analysis, all of which are parameters which may indicate a (forme fruste) keratoconus. Densitometry was only significantly different in the 6–10 mm zone and not in the central zone as is typical for keratoconus (Lopes et al. 2014). Limitations of the study are that the age of the patients and controls may be too young for progression to keratoconus to have taken place, the small number of patients and the lack of genetic analysis (Keleş et al. 2020).

Several other studies and case reports may possibly show a link between OI and the development of keratoconus (Evereklioglu et al. 2002; Kwitko & Pretto 2017; Zeri et al. 2017; Koyun et al. 2018).

Zimmermann et al. (1988) investigated the difference in collagen composition of normal corneas and corneas with keratoconus. In one case with OI they found decreased amount of collagen type I.

Karimian et al. (2014) described six patients with keratoglobus, one of which was diagnosed with OI. All patients had limbal stem cell‐sparing keratoplasty.

#### 2. Refractive errors

Refractive errors were reported in 18 articles (Table 4) (Superti‐Furga et al. 1989; Madigan et al. 1994; Evereklioglu et al. 2002; Eliott et al. 2003; Scott et al. 2005; Barbirato et al. 2009; Benzimra et al. 2012; Rishi et al. 2012; Salcone et al. 2014; Wallace et al. 2014; Bohnsack 2016; Sendul et al. 2016; Klug & Bek 2017; Kwitko & Pretto 2017; McVey et al. 2017; Zeri et al. 2017; Bellanca et al. 2018; Campagna et al., 2018). The majority of these articles reported refractive errors as an additional finding in different types of OI and at different ages. Myopia was the most reported refractive error (Superti‐Furga et al. 1989; Madigan et al. 1994; Eliott et al. 2003; Scott et al. 2005; Barbirato et al. 2009; Rishi et al. 2012; Salcone et al. 2014; Wallace et al. 2014; Bohnsack 2016; Klug & Bek 2017; Kwitko & Pretto 2017; McVey et al. 2017; Zeri et al. 2017; Bellanca et al. 2018; Campagna et al., 2018). Severe myopia (>−6D) was seen in six cases (Eliott et al. 2003; Rishi et al. 2012; Wallace et al. 2014; Zeri et al. 2017; Bellanca et al. 2018; Campagna et al., 2018). Axial length (AL) of the globe was only measured in three patients (Scott et al. 2005; Bohnsack 2016; Bellanca et al. 2018). Bellanca et al. (2018) showed an axial length of 30.00 mm OD and 29.50 mm OS with a refractive error of −7.5D and −6.75D respectively. The cause of myopia in the articles in which the AL was not measured could also be due to high keratometry values. Kaiser‐Kupfner, as mentioned above, reported shorter AL in OI patients compared to control patients. Zeri et al. (2017) reported a patient with high myopia caused by keratoconus. Retinal detachment in a patient with severe myopia was described by Eliott et al. (2003). Koyun et al. (2018) reported −0.5D (SD ±1.2D) as mean values for spherical equivalent in a group of 15 children with OI (type I and IV).

**Table 4:** Refractive errors in OI patients

Article	Gender (M/F)	OI type	Age (Y/M)	Scleral hue	Refraction (D)	Axial length (mm)
Superti et al. 1989	M	IV	19 M	Blue	‐	‐
	F	IV	28Y	Light blue	Myopia	‐
	F	IV	66Y	Blue	Severe myopia	‐
	M	IV	82Y	Grey	Myopia	‐
	F	IV	48Y	Grey	Myopia	‐
	M	IV	42Y	Grey	Severe myopia	‐
Madigan et al. 1994	F	‐	13Y	Blue	−1.0/−1.0	‐
Evereklioglu et al. 2002, *	M/F	I/IV	10.1 ± 2.5Y	‐	Myopia	‐
Eliott et al. 2003	M	‐	57Y	Blue	−11.0 OU	‐
	M	‐	61Y	Blue	−2.0/−5.25	‐
	M	‐	54Y	Blue	‐	‐
Scott et al. 2005	F	I	40Y	Blue	−2.5/−2.5 OS to −4.0	24.96/25.64
Barbirato et al. 2009	M	III	16Y	Blue	Myopia	‐
Benzimra et al. 2012	F	I	73Y	Blue	+7.0/+6.5	‐
Rishi et al. 2012	F	‐	12Y	Blue	−11.0/−18.0	‐
Salcone et al. 2014	F	I	37Y	Blue	Low myopia	‐
Wallace et al. 2014	F	I	74Y	Blue	+1.75/+2.25	‐
	F	I	70Y	Blue	+0.5/+0.75	‐
	F	I	49Y	‐	−10.75/−5.75	‐
Bohnsack 2016	M	‐	6M	Blue	+0.75/−5.5	21.4/24.3
Sendul et al. 2016	F	‐	12Y	Blue	+3.5/+5.0	‐
Klug & Bek 2017	F	‐	19Y	White	−1.0 OS	‐
Kwitko & Pretto 2017	F	‐	6Y	‐	−1.5/−1.75 5 months later −3.0/−3.0	‐
McVey et al. 2017	M	‐	37Y	Blue	Myopia	‐
	M	‐	19 M	Blue	‐	‐
Zeri et al. 2017	M	I	65Y	Blue	+2.25/+1.75	‐
	F	I	31Y	Blue	−9.5/−10.0	‐
	F	I	4Y	Blue	−2.5/−2.5	‐
Bellanca et al. 2018	M	I	28Y	Blue	−7.5/−6.75	30.00/29.5
Campagna et al. 2018	M	I	12Y	White	−18.25/−14.75	‐

M = male, F = female, Y = years, M = months.

* 5 of 23 patients reported with myopia, OI type and age of specific patients not reported.

#### 3. Sclera

##### 3a. Blue sclera

Blue sclera is a characteristic finding and often the first symptom of OI in children. This systematic review showed blue sclera to be the most frequent reported finding in patients with OI. Furthermore, in 48 articles it is the only ocular finding (Sillence et al. 1979; Beighton 1981; Crawfurd & Winter 1982; Paterson et al. 1983; Lanting et al. 1985; Paterson et al. 1987; Brooks et al. 1989; Wenstrup et al. 1990; Garretsen & Cremers 1991; Mottes et al. 1992; Marion & Hinojosa 1993; Sillence et al. 1993; Superti‐Furga et al. 1993; Cherie‐Ligniere et al. 1995; Leidig‐Bruckner & Grauer 1998; Heimert et al. 2002; Benusiené & Kucinskas 2003; Saeed et al. 2003; Hazenberg & Bom 2005; Liu et al. 2007; Barbirato et al. 2009; Bhadada et al. 2009; Lin & Lin 2009; Rauch et al. 2010; Aftab et al. 2013; Balasubramanian et al. 2013; Chatterjee et al. 2013; Niramitmahapanya et al. 2013; Ren et al. 2014; Brizola et al. 2015; Cho et al. 2015; Fan et al. 2015; Lindahl et al. 2015; Zhang et al. 2016; Ackermann & Levine 2017; Aslan et al. 2017; Binh et al. 2017; Brizola et al. 2017; Liu et al. 2017; McVey et al. 2017; Costantini et al. 2018; Mitaka 2018; Devadas 2019; Kimura et al. 2019; Maioli et al. 2019; Zhytnik et al. 2019; Aissaoui et al. 2020; Udomchaiprasertkul et al. 2020). Blue sclera is described predominantly in OI type I and type IV. Sillence et al. (1993) reported a significant difference in scleral hue between type I and type III/IV patients, blue sclera being more frequent in type I patients. The sclera of patients with OI type III or IV can be blue at birth, but the intensity of the colour fades away as they age. Blue sclera is also present in some patients with OI type V. A recent case‐series by Brizola et al. (2015) described the finding of blue sclera in two of their seven patients with OI type V. The study by Kaiser‐Kupfer et al., (1985a, 1985b) showed a correlation between low ocular rigidity and blue sclera.

##### 3b. Staphyloma

Scott et al. (2005) described in a case report a 40‐year‐old woman with OI type I and blue sclera. She had atrophic signs of myopia. Optical coherence tomography (OCT) showed a posterior staphyloma of the left eye.

#### 4. Retina

Retinal detachment was described in five articles (Madigan et al. 1994; Eliott et al. 2003; Church & Winder 2006; Jonisch & Deramo 2011; Fleissig & Barak 2019). Eliott et al. (2003) described three middle aged patients with retinal detachment. One of the patients had high myopia and retinal detachment in both eyes. All patients were successfully treated. However, in one of the patients vitrectomy was complicated by the collapse of the globe. Fleissig et al. and Church et al. both described a patient (OI type III) with a retinal detachment at a relatively young age (37 and 31 years). Both patients were treated with success (Church & Winder 2006; Fleissig & Barak 2019). Madigan et al. (1994) presented the case of a child of 13 years old with a retinal detachment of the left eye, found on routine ocular examination. She was successfully treated with pneumatic retinopexy. All these articles mentioned the thinness of the sclera as a complicating factor during treatment.

#### 5. Bruch’s membrane/choroidea

Choroidal neovascularization was mentioned in four case‐reports, all of which described one patient (Rishi et al. 2012; Klug & Bek 2017; Zeri et al. 2017; Bellanca et al. 2018). Three patients presented with unilateral sudden visual loss and metamorphopsia. Clinical examination revealed choroideal neovascularization caused by a lacquer crack. The patient described by Bellanca et al. (2018) showed spontaneous improvement of the symptoms after one‐month follow‐up. The other two patients by Rishi et al. (2012) and Klug et al. (2017) were treated with intravitreal anti‐VEGF injections resulting in decreased symptoms. Zeri et al. (2017) described a 31‐year‐old patient with OI type I who presented with a neovascular membrane in the right macula at the age of 27, for which she received photodynamic therapy. In all patients symptoms occurred at a relatively young age (28, 12, 19 and 27 years). Three of the patients had high myopia, which is a risk factor for choroidal neovascularization (Rishi et al. 2012; Zeri et al. 2017; Bellanca et al. 2018).

#### 6. Intraocular pressure and Glaucoma

##### 6a. Intraocular pressure

Lagrou et al. (2017) reported decreased corneal hysteresis (CH), corneal resistance factor and CCT in ten children with OI, presumably leading to underestimation of the eye pressure measured by applanation tonometry. Tonometry IOP was significantly decreased in OI, but after calculating the corneal‐compensated IOP this was significantly higher in OI compared to controls.

##### 6b. Primary open‐angle glaucoma

Five articles were found describing glaucoma in patients with OI (Superti‐Furga et al. 1989; Wallace et al. 2014; Bohnsack 2016; Mauri et al. 2016; Laroche & Nkrumah 2020). The paper by Wallace et al. (2014) described three patients (from two families) with OI type I and primary open‐angle glaucoma (POAG). Central corneal thickness (CCT) measurements were lower in the affected patients compared to healthy controls. Genetic testing of *MYOC,* a known glaucoma‐associated gene, was performed but no mutations were found in any of the patients. Laroche & Nkrumah (2020) reported on a 57‐year‐old woman with POAG. She had a medical history of cataract surgery and prior selective laser trabeculoplasty. IOP reduction with medication was not successful, but microinvasive glaucoma surgery led to reduction of IOP.

##### 6c. Primary congenital glaucoma and infantile‐onset glaucoma

Mauri et al. performed *COL1A1* analysis in a group of 27 patients with primary congenital glaucoma (PCG) or early‐onset glaucoma. A *COL1A1* mutation was found in four patients with variable clinical symptoms of OI. The authors suggest genetic screening for *COL1A1* mutations in patients with PCG/early‐onset glaucoma and advise ophthalmologic screening for glaucoma in OI patients with a known *COL1A1* mutation (Mauri et al. 2016). Bohnsack et al. (2016) presented a 6‐month old boy with OI and bilateral buphthalmos with corneal oedema in the left eye. The left eye was successfully treated with a trabeculotomy. Follow‐up showed no evidence of glaucoma progression and excellent visual behaviour.

#### 7. Trauma

##### 7a. Accidental

Five case reports described ruptures or laceration of the cornea after minor trauma in young patients with OI type I (Natarajan et al. 2003; Unver et al. 2009; Polat & Ulucan 2015; Oh et al. 2016; Campagna et al., 2018). Two case reports reported a corneal laceration in a child (Natarajan et al. 2003; Campagna et al., 2018). Three case reports described corneal or globe ruptures caused by finger trauma or eye‐rubbing (Unver et al. 2009; Polat & Ulucan 2015; Oh et al. 2016). All studies reported fragility of the cornea which made surgical repair more difficult. Scleral rupture was described by Pirouzian et al. (2007) in three children, between 5 and 15 years of age, due to scleral fragility.

Retinal haemorrhages after a fall from low height were described in three patients with OI type I by Ganesh et al. (2004). All patients were of a very young age (7–18 months). A CT‐scan of the brain showed an acute subdural hematoma in all three patients. Due to the presence of retinal haemorrhages, investigation of child abuse took place in all three cases, but could be ruled out.

##### 7b. Complications in elective surgery

Salcone et al. (2014) described a 37‐year‐old patient with diplopia who underwent strabismus surgery which was complicated by a full thickness scleral perforation. Upon suturing of the perforation the sclera was noted to be soft and two days later a second small wound adjacent to the first was discovered. Closing required a scleral donor patch. The patient underwent a second successful strabismus surgery on the right eye two years later.

### Incidental findings

#### 1. Strabismus

Andalib et al. (2009) published a case‐report of a 2‐year‐old girl with OI who was referred to the hospital at 6 months of age due to ocular deviation and ptosis in the left eye. She was diagnosed with a nervus oculomotorius palsy for which she underwent successful surgery.

#### 2. Anterior segment

##### 2a. Absence of Bowman’s layer

Kasner et al. (1992) described in a post‐mortem study a patient with OI type III without a Bowman’s layer. The absence of Bowman’s layer was not associated with any evidence of scarring or inflammation, which could conclude that there was a congenital absence of Bowman’s layer. Kobayashi et al. (2014) also reported two patients (OI type I) with thin corneas, an absent or atrophic Bowman’s layer and K‐structures – usually seen between Bowman’s layer and subepithelial stroma – were completely absent.

##### 2b. Detachment of Descemet membrane

Two studies described detachment of the Descemet membrane (Gorovoy et al. 2012; Polat & Ulucan 2015). Gorovoy et al. (2012) described a 25‐year‐old man who presented with acute loss of vision in his right eye due to a totally detached and taut Descemet membrane. Attempt to reattach the Descemet membrane with air bubbling was unsuccessful. The patient was successfully treated with Descemet Stripping Automated Endothelial Keratoplasty (DSAEK).

##### 2c. Anterior Chamber

Nwosu et al. (2005) described a 19‐year‐old patient with Type I OI who was diagnosed with Rieger’s anomaly with corneal epithelial oedema and iris atrophy in both eyes. This patient also had overlapping features of Ehlers‐Danlos syndrome.

#### 3. Posterior segment

##### 3a. Retinal artery occlusion

A 12‐year‐old girl with OI type I, underwent surgery for correction of scoliosis. This surgery was performed under hypotensive anaesthesia and the patient was placed in a prone position. Shortly after this surgery the patient developed loss of vision of the whole visual field in her left eye, due to a retinal artery occlusion. Treatment with intravenous Acetazolamide and external ocular massage was unsuccessful (Bradish & Flowers 1987). In general, ocular artery occlusion is a known possible complication after hypotensive anaesthesia.

##### 3b. Macula

Benzimra et al. (2012) described a 73‐year‐old patient (OI type I) with reduced central vision of the right eye for two months. Vision of the left eye was poor since childhood. Examination revealed full thickness macula holes in both eyes. Phaco‐vitrectomy was successful and without complications in both eyes.

Sendul et al. (2016) described a 12‐year‐old girl in which routine eye examination revealed unexpected eye pathologies. Fundus examination showed stage 2 papilloedema in both eyes, a macular pucker on OCT and thickening in the retinal nerve fibre layer in both eyes. Whilst the papilloedema regressed spontaneously, thinning of the retinal nerve fibre layer and bitemporal partial hemianopia remained.

##### 3c. Retinoblastoma

Haik (1984) described three family members with retinoblastoma. One patient, a 16‐month‐old boy, was also diagnosed with OI. Surgical removal of the right eye was performed. Follow‐up till the age of 30 has been uneventful. Unfortunately further clinical information about the status of his OI is lacking.

## Discussion

The purpose of the current paper was to systematically review the existing literature describing eye problems in patients diagnosed with OI. To the best of our knowledge, this is the first systematic review which covers ocular problems occurring in patients with OI. The results of this review show a wide range of eye problems that affect almost every component of the eye. The most frequently described ocular findings in OI involve blue sclera, myopia, thinning of the cornea, traumas and the risk of developing glaucoma.

The sclera and cornea seem to be the most affected parts of the eye in patients with OI. Type I collagen is an important structural component of the cornea as well as of the sclera, so it can indeed be expected that many eye problems occur in these tissues (Meek & Fullwood 2001). Several studies showed decreased central corneal and scleral thickness in OI patients compared to healthy controls. Weakening of these structures may be the cause of several problems, such as blue discoloration of the sclera and rupture of the globus after a minor trauma.

The cornea is the source of most of the refractive power of the eye, and alterations of the cornea could affect the eye’s refractive properties. Myopia is a common finding in patients with OI. The fragility of the sclera could subsequently result in the development of retinal tears and retinal detachment. Patients seem more susceptible to retinal haemorrhages resulting from minor trauma. As several studies in this review showed, routine ophthalmological procedures could result in unexpected complications and therefore ophthalmic surgery in patients with OI should be carefully considered and approached with caution.

Thinning of the cornea could potentially lead to the development of keratoconus. However, the published studies by Magalhaes et al. (2018) and Keleş et al. (2020) showed no direct correlation between OI and increased risk of developing keratoconus. Further research is required because both studies have several limitations such as a limited number of patients and young age of the patients.

Elevated IOP is a major risk factor for glaucoma. The significantly lower central corneal thickness in OI patients, as pointed out by Lagrou et al. (2017), is a risk factor for underestimation of IOP. Therefore, when measuring a normal IOP in patients with OI, special attention should be given to the aspect of the optic disc, signs of glaucomatous cupping, the retinal nerve fibre layer thickness, and visual field testing. Both corneal thickness and biomechanical properties should be taken into account and a lower threshold should be considered for treating glaucoma in this patient population. Low central corneal thickness is associated with a higher risk of open‐angle glaucoma independent of IOP, which may also apply in this population. Decreased amounts of collagen type I in the sclera are accompanied by alterations in the trabecular meshwork which could potentially increase the aqueous outflow resistance leading to an increase in IOP (Watson & Young 2004).

This review showed that OI patients have an increased risk of corneal and scleral ruptures after minor trauma. In both the cornea and the sclera, alterations in collagen composition lead to decreased rigidity of the structures. Together with the decreased corneal hysteresis, they make the OI eyes more sensitive to the transmission of forces into the eye resulting in ruptures and lacerations after minor trauma. Especially in young children this is of great concern as even rubbing of the eye could lead to rupturing. It is therefore recommended that OI patients wear protective glasses to prevent accidental eye trauma. Younger children especially, could benefit from wearing these glasses, and both children and adults should consider them when playing sports or engaging in other physical activities. It is important for patients and parents to be aware of the potential risk of eye problems in OI. Education regarding alarm symptoms and when to contact an ophthalmologist could be beneficial. However, current literature does not seem to indicate the need for annual check‐ups with an ophthalmologist in patients without symptoms.

Based on the findings in this review, no statement can be made about the prevalence of eye problems in patients with OI. Most patients described in this review were diagnosed with OI type I, the most prevalent type of OI. Some patients suffered from multiple eye problems which were related to each other. More research is required to obtain a better understanding of which eye problems are directly related to OI, and which eye problems are only coincidental. The knowledge about the pathophysiological changes in collagen of the eye in OI is still unknown. Future research on the structure and pathophysiological changes of the collagen fibres in the eyes of patients with OI is required.

Several limitations of this review should be taken into consideration. Our literature search resulted in limited numbers of published studies and patients, which makes it difficult to find an association between type of OI and the occurrence of certain eye abnormalities. For this review, only patients with OI types I–V were included, so there is a possibility we missed interesting articles describing eye problems in patients with other types of OI. It was not always possible to correlate eye problems with the type of OI, given that in 35% of the articles the type of OI was unknown or uncertain. Considering that collagen type I is central to the pathology of the disease, it would be interesting to relate eye anomalies to molecular defects. However, many of these articles date several years back when genetic testing was not performed or widely available. We decided to include articles based on the described clinical findings, and they were excluded if they did not match the characteristics for OI Type I–V. Another limitation of this review is that only articles described in English or Dutch have been included. By excluding articles written in other languages, this review does not represent the full picture of all eye defects related to OI; as a result, the actual number of eye problems that occur in patients with OI could be underestimated. Furthermore, as a result of the high number of case reports or studies with a low number of patients, and the wide ranges of age groups and differences in demographic profiles and OI types, findings in some studies may not have enough power to draw correlations. It is therefore advised that future studies focus more on cohort and case–control studies with an increased number of patients and with extensive clinical and molecular characterization. Nonetheless, despite the relatively limited number of patients, this review offers valuable insights into possible eye problems in patients with OI.

In summary, this systematic review attempts to stress the importance of taking eye examinations into consideration in OI patients. The cornea and sclera are mainly composed of type I collagen and it has been shown that these parts of the eye seem to be thinner and more fragile in patients with OI. Due to the thinness of these structures several aspects should be considered: underestimation of IOP could occur, protective glasses should be worn to reduce the risk of accidental eye trauma, and ocular surgery should be performed with great caution because of the increased risk of complications. Osteogenesis imperfecta (OI) patients need to be made aware of the potential issues that could occur when they have been diagnosed with OI. This review also tries to emphasize the requirement for intensive research on the underinvestigated ocular complications in OI. This can potentially contribute to improvement of patient care guidelines and hopefully stimulate research towards finding new ways to address the underlying pathology.

## Supporting information

Supplementary information search strategy.
