# Supplementary material for: Ocular characteristics and complications in patients with osteogenesis imperfecta: a systematic review
Source: Acta Ophthalmol. 2021 May 19;100(1):e16–28. doi: 10.1111/aos.14882 (PMC9290710; doi:10.1111/aos.14882)
Supplement: Supplementary file 1 — Supplementary information search strategy. [file AOS-100-e16-s001.docx]

**Supplementary information search strategy**

**PubMed Session Results (08 Aug 2020)**

| Search | Query | Items found |
| --- | --- | --- |
| #3 | **#1 AND #2** | 584 |
| #2 | **"Ocular Physiological Phenomena"[Mesh] OR "Eye"[Mesh] OR "Eye Diseases"[Mesh] OR "Visually Impaired Persons"[Mesh] OR "eye"[tiab] OR "eyes"[tiab] OR "orbital*"[tiab] OR "intraorbital*"[tiab] OR "ocular*"[tiab] OR "retina*"[tiab] OR "cornea*"[tiab] OR "sclera*"[tiab] OR "visual impair*"[tiab] OR "vision disab*"[tiab] OR "vision impair*"[tiab] OR "visual disab*"[tiab] OR "visually impair*"[tiab] OR "visually disab*"[tiab]** | 1,055,675 |
| #1 | **"Osteogenesis Imperfecta"[Mesh] OR "osteogenesis imperfect*"[tiab] OR "brittle bone*"[tiab] OR "Lobstein*"[tiab] OR "Fragilitas Ossium*"[tiab] OR "osteopsathyros*"[tiab]** | 5,965 |

**Embase.com Session Results (08 Aug 2020)**

| Search | Query | Items found |
| --- | --- | --- |
| #3 | **#1 AND #2** | 1,344 |
| #2 | **'visual system function'/exp OR 'eye disease'/exp OR 'eye'/exp OR 'visually impaired person'/exp OR eye*:ab,ti,kw OR orbital*:ab,ti,kw OR intraorbital*:ab,ti,kw OR ocular*:ab,ti,kw OR retina*:ab,ti,kw OR cornea*:ab,ti,kw OR sclera*:ab,ti,kw OR 'vision disab*':ab,ti,kw OR 'vision impair*':ab,ti,kw OR (visual* NEXT/3 impair*):ab,ti,kw OR (visual* NEXT/3 disab*):ab,ti,kw** | 1,625,799 |
| #1 | **'osteogenesis imperfecta'/exp OR 'osteogenesis imperfecta tarda'/exp OR 'osteogenesis imperfecta case reports'/exp OR 'osteogenesis imperfecta congenita'/exp OR 'osteogenesis imperfecta complications'/exp OR 'osteogenesis imperfecta type i'/exp OR 'osteogenesis imperfecta type 1'/exp OR 'osteogenesis imperfecta type iii'/exp OR 'osteogenesis imperfect*':ab,ti,kw OR 'brittle bone*':ab,ti,kw OR Lobstein*:ab,ti,kw OR 'Fragilitas Ossium*':ab,ti,kw OR osteopsathyros*:ab,ti,kw** | 8,534 |

**Scopus Session Results (08 Aug 2020)**

| Search | Query | Items found |
| --- | --- | --- |
| #3 | **#1 AND #2** | 494 |
| #2 | **TITLE-ABS (eye* OR orbital* OR intraorbital* OR ocular* OR retina* OR cornea* OR sclera* OR "vision disab*" OR "vision impair*" OR (visual* PRE/3 impair*) OR (visual* PRE/3 disab*)) OR AUTHKEY (eye* OR orbital* OR intraorbital* OR ocular* OR retina* OR cornea* OR sclera* OR "vision disab*" OR "vision impair*" OR (visual* PRE/3 impair*) OR (visual* PRE/3 disab*))** | 1,181,566 |
| #1 | **TITLE-ABS ("osteogenesis imperfect*" OR "brittle bone*" OR Lobstein* OR "Fragilitas Ossium*" OR osteopsathyros*) OR AUTHKEY ("osteogenesis imperfect*" OR "brittle bone*" OR Lobstein* OR "Fragilitas Ossium*" OR osteopsathyros*)** | 6,244 |
